# Thyroid Hormone Plays an Important Role in Cardiac Function: From Bench to Bedside

**DOI:** 10.3389/fphys.2021.606931

**Published:** 2021-10-18

**Authors:** Hiroyuki Yamakawa, Tomoko S. Kato, Jaeduk Yoshimura Noh, Shinsuke Yuasa, Akio Kawamura, Keiichi Fukuda, Yoshiyasu Aizawa

**Affiliations:** ^1^Department of Cardiology, Keio University School of Medicine, Tokyo, Japan; ^2^Center for Preventive Medicine, Keio University School of Medicine, Tokyo, Japan; ^3^Department of Cardiology, International University of Health and Welfare Narita Hospital, Chiba, Japan; ^4^Department of Internal Medicine, Ito Hospital, Tokyo, Japan

**Keywords:** thyroid hormone, hyperthyroidism, hypothyroidism, cardiovascular disease, genomic pathways, non-genomic pathways, amiodarone, cardiac regeneration

## Abstract

Thyroid hormones (THs) are synthesized in the thyroid gland, and they circulate in the blood to regulate cells, tissues, and organs in the body. In particular, they exert several effects on the cardiovascular system. It is well known that THs raise the heart rate and cardiac contractility, improve the systolic and diastolic function of the heart, and decrease systemic vascular resistance. In the past 30 years, some researchers have studied the molecular pathways that mediate the role of TH in the cardiovascular system, to better understand its mechanisms of action. Two types of mechanisms, which are genomic and non-genomic pathways, underlie the effects of THs on cardiomyocytes. In this review, we summarize the current knowledge of the action of THs in the cardiac function, the clinical manifestation and parameters of their hemodynamics, and treatment principles for patients with hyperthyroid- or hypothyroid-associated heart disease. We also describe the cardiovascular drugs that induce thyroid dysfunction and explain the mechanism underlying the thyroid toxicity of amiodarone, which is considered the most effective antiarrhythmic agent. Finally, we discuss the recent reports on the involvement of thyroid hormones in the regulation of myocardial regeneration and metabolism in the adult heart.

## Introduction

The thyroid gland secretes two thyroid hormones (THs), 3,5,3′-triiodothyronine (T3) and 3,5,3′,5′−tetraiodothyronine (T4 also known as thyroxine). Moreover, THs are synthesized using iodine, influence metabolism, and biosynthesize proteins in the body. These THs are regulated by thyroid stimulating hormone (TSH), which is secreted by the anterior pituitary gland. In turn, TSH is regulated by the hypothalamus via thyrotropin-releasing hormone (TRH). Thyroid hormones exhibit a variety of effects on the heart and peripheral vascular system It is well known that they raise the heart rate and cardiac contractility, improve the systolic and diastolic function of the heart, and decrease the systemic vascular resistance (SVR) in resting condition (Klein and Ojamaa, 2001).

Thyroid dysfunction, which causes hyperthyroidism and hypothyroidism, is associated with increased cardiovascular risk factors (Klein and Ojamaa, 2001; Rodondi et al., 2010; Collet et al., 2012). For example, hyperthyroidism increases the risk of atrial fibrillation (AF), cardiovascular disease (CVD), and heart failure (HF) (Biondi, 2012). Conversely, hypothyroidism is associated with hypertension and dyslipidemia and also causes CVD (Rodondi et al., 2010; Pearce, 2012). In particular, the intracellular effects of THs in cardiomyocytes occur via two types of mechanisms, genomic and non-genomic, with the genomic pathway predominating (Khan et al., 2020). The details of the mechanisms are described in Section 3.

There has been a long controversy regarding whether increased cardiovascular risk is related to thyroid dysfunction (Langén et al., 2018) and whether there is an association between thyroid disorder and the risk of sudden cardiac death (SCD) (Chaker et al., 2016). Sudden cardiac death is an unexpected death or arrest from a cardiovascular cause that occurs outside a hospital or in the emergency room (Lopshire and Zipes, 2006). The major cause of SCDs is lethal ventricular arrhythmias in patients with underlying coronary heart disease (Weisfeldt et al., 2011; Hayashi et al., 2015). Moreover, SCD also develops within 1 h. Over 50% of SCD cases are due to coronary hearts diseases (Hayashi et al., 2015), and these account for almost 20% of the total mortality.

Several groups have reported a relationship between thyroid function and SCD. Charker et al. concluded that elevated free T4 levels might increase the risk of SCD in patients with euthyroid thyroid disease, studied in a prospective population-based cohort (Chaker et al., 2016). Mitchell et al. investigated whether patients with HF with a reduced ejection fraction (HFrEF) and a thyroid functional disorder had an increased risk of SCD (Mitchell et al., 2013). They concluded that a thyroid dysfunction in patients with symptomatic HF and an ejection fraction ≤ 35% had a strong positive correlation with risk of death. Similar results were obtained after adjusting for known mortality predictors [Sudden Cardiac Death in Heart Failure Trial (SCD-HeFT)] (Mitchell et al., 2013). Langén et al. reported that thyroid dysfunction could be related to an increased overall mortality and risk of SCD and that large-scale randomized control trials are essential to decide whether to treat patients with mild thyroid insufficiency (Langén et al., 2018).

In this review, we summarize the effects of TH on the heart (Klein and Ojamaa, 2001) and the clinical symptoms of thyroid dysfunction from the viewpoint of the cardiology (Klein and Danzi, 2007). In addition, we discuss the changes in and the mechanisms of TH metabolism that have an influence on arrhythmias and congestive HF (Danzi and Klein, 2014). Further, we specify the cardiovascular drugs that induce thyroid dysfunction and explain the mechanism underlying the thyroid toxicity of amiodarone, which is considered the most effective antiarrhythmic agent.

## Effects of Thyroid Hormones on the Cardiovascular System

In the thyroid gland, two main iodinated hormones, T3 (triiodothyronine) and T4, (tetraiodothyronine; also known as thyroxine) are secreted. By binding to thyroid hormone receptors (TRs), T3 and T4 exert biological activity in responsive tissues. T3 is regarded as a biologically active hormone (Jabbar et al., 2017), and T4 has a few documented non-genomic effects but is largely regarded as a prohormone. Most T4 is deiodinated to T3 in the liver, kidneys, and skeletal muscle (Klein and Danzi, 2007). T3 is carried through blood circulation to each target tissue and organ such as the heart and peripheral blood vessels. Then, these tissues and organs are regulated by serum levels of T3 solely or preponderantly (Danzi and Klein, 2014).

Symptoms of hyperthyroidism include cardiac and hemodynamic symptoms, such as palpitations, widened pulse pressure, dyspnea on exertion, tachycardia, exercise intolerance, and AF (Table 1) (Dahl et al., 2008). Cardiac contractility, as well as resting heart rate, is increased by THs. Cardiac output can increase by 50–300% under hyperthyroidism compared to that in normal conditions. This enhancement of cardiac output is based on synergistic effects of a raised heart rate, increased cardiac contractility, and dilation of peripheral blood vessels (Biondi et al., 2002).

**TABLE 1:** Cardiovascular clinical manifestations and laboratory findings associated with hyperthyroidism and hypothyroidism (Danzi and Klein, 2014).

Hyperthyroidism				
Palpitations	Anginal chest pain	Exercise intolerance	Atrial fibrillation	Cardiac hypertrophy
Systolic hypertension	Peripheral edema	Hyperdynamic precordium	Pulmonary hypertension	Heart failure

**Hypothyroidism**				

Fatigue	Decreased endurance	Increased serum cholesterol	Impaired cardiac contractility	Increased SVR
Bradycardia	Decreased endothelial-derived relaxation factor	Increased homocysteine	Increased C-reactive protein	

*SVR, systemic vascular resistance.*

The cardiovascular symptoms of hypothyroidism are not obvious, in contrast to the distinct clinical manifestations of hyperthyroidism. Multiple characteristic phenotypes of hypothyroidism have been described, including bradycardia, diastolic hypertension, narrow pulse pressure, fatigue, myalgia, elevated cholesterol, and a feeling of puffiness (Table 1), but the serum TSH level is an accurate diagnostic indicator of hypothyroidism (Dahl et al., 2008). The cardiovascular effects of hypothyroidism significantly differ from those of hyperthyroidism; for example, cardiac output can be reduced by 30 to 50%, compared to that in a normal state (Danzi and Klein, 2004). However, it is significant that the treatment of hypothyroidism can normalize cardiovascular hemodynamics with a slight change in the resting heart rate (Crowley et al., 1977).

## Mechanisms of Thyroid Hormone Effects at the Cellular Level

### Mechanisms Underlying the Intracellular Cardiac Effects of Thyroid Hormones

The effects of THs at the cardiac intracellular level are divided into genomic and non-genomic pathways (Khan et al., 2020). In the genomic pathway, THs regulate the expression of target genes by binding to nuclear receptors in cardiomyocytes. In contrast, the non-genomic pathway includes effects on ion channels of the cardiomyocytes and effects of THs on the peripheral circulation, which regulate hemodynamics and the cardiac ejection fraction (Klein and Ojamaa, 2001; Cooper and Biondi, 2012).

### Genomic Response to Thyroid Hormones

Thyroid hormones (THs) regulate the expression of genes coding for cardiac proteins. T3 binds to TRs in the cardiomyocyte nucleus, and this regulates transcription by binding to thyroid hormone response elements (TREs) in regulatory regions of target genes (Kahaly and Dillmann, 2005). Thyroid hormone responses (TRs) are members of the superfamily of steroid hormone receptors. An important feature of their activity is that they bind to TREs with or without ligand, which is distinct from other steroid hormone receptors. TRs bind to TREs as retinoid X receptors (RXRa, RXRb, or RXRg) (Lazar and Chin, 1990; Giammanco et al., 2020).

Table 2 shows the regulation by TH of genes coding for cardiac proteins (Klein and Ojamaa, 2001). One effect of TH in cardiomyocytes is to control cardiac contractility and ejection fraction. THs upregulate the expression of genes encoding sodium/potassium-transporting ATPases (Na^+^/K^+^ ATPase), α-myosin heavy chain (myosin heavy chain 6; encoded by *MYH6*), and sarcoplasmic/endoplasmic reticulum calcium ATPase 2 (SERCA2; encoded by *ATP2A2*) and downregulate the transcription of β−myosin heavy chain (myosin heavy chain 7; encoded by *MYH7*) and phospholamban (PLN; encoded by *PLN*) (He et al., 1997; Kaasik et al., 1997; Holt et al., 1999).

**TABLE 2:** Regulation of the genes coding for cardiac proteins by THs [modified from reference (Klein and Ojamaa, 2001)].

	Upregulate	Downregulate
Myofilament		
	α-MHC (*MYH6*)	β-MHC (*MYH7*)
Ca handling protein		
	SERCA (*ATP2A*2)	Phosphlamban (*PLN*)
Membrane channel		
	Na^+^/K^+^ ATPase	Na^+^/Ca^2+^ exchanger
Adrenergic receptor pathway		
	b1 Adrenergic receptor	Adenylyl cyclase type V, VI
Fibrosis		
	MMP2, TIMP1-4	
Voltage-gated potassium channels		
	Kv1.5, Kv4.2, Kv4.3	

α-myosin and β-myosin heavy chains are major components of the cardiomyocyte contractile structures called sarcomeres (Nadal-Ginard and Mahdavi, 1989). The upregulation of SERCA2 and the downregulation of PLN increase calcium concentrations in cardiomyocytes and enhance systolic contraction. Thyroid hormones can increase the protein expression of SERCA2 and decrease the protein expression of PLN in the sarcoplasmic reticulum, leading to improved ventricular relaxation (Kiss et al., 1994; Kranias and Hajjar, 2012). Thyroid hormones (especially T3) also have a direct inotropic effect on cardiomyocytes by upregulating the expression of the β1-adrenergic receptors (Hoit et al., 1997). On the other hand, T3 has been reported to suppress the expression of adenylate cyclase (Klein and Danzi, 2007, 2016). In another study, the authors hypothesized that T3 primarily regulates genes that control cardiac pacemaker cells, exerting a positive chronotropic effect in these cells (Klein and Danzi, 2016).

Chen et al. reported that treatment with a T4 antagonist decreases the collagen fibers in the left ventricular non-infarcted area, with increasing expression of matrix metalloproteinase-2 (MMP2) and tissue inhibitor of matrix metalloproteinases 1 to 4 (MMP1 to 4). Paradoxically, atrial fibrosis is also decreased by T4 (Chen et al., 2013; Zhang et al., 2014), and stimulation by various THs increased cardiac angiogenesis as well as cardiomyocyte growth (von Hafe et al., 2019). In addition, THs regulate several plasma membrane and ion transporters, for example Na^+^/K^+^-ATPase, Na^+^/Ca^2+^ exchanger (NCX) and voltage-gated potassium channels, including Kv1.5, Kv4.2, and Kv4.3, at both the transcriptional and post-transcriptional levels. Consequently, THs regulate cardiomyocyte excitation-contraction coupling (EC coupling) responses (Gick et al., 1990; Ojamaa et al., 1999).

### Non-genomic Response to Thyroid Hormone

Thyroid hormones (THs) have two major non-genomic effects, on cardiomyocytes of several membrane ion channels, such as Na^+^, K^+^, and Ca^2+^ channels (Klein and Ojamaa, 2001; Giammanco et al., 2020). In neonatal rat cardiomyocytes, they regulate phosphatidylinositol 3-kinase (PI3K) or serine/threonine-protein kinase (AKT) signaling pathways (Kuzman et al., 2005). Thyroid hormone prevents cell death by AKT pathway in the vascular smooth muscle (Ojamaa et al., 1996).

Other than these pathways, PI3K/AKT-induced physiological hypertrophy is regulated by insulin-like growth factor-1 (IGF-1) (Fujio et al., 2000), p85α (Kenessey and Ojamaa, 2006), angiotensin-1 receptor (Diniz et al., 2009), ubiquitin proteasomes (Rajagopalan et al., 2013), epidermal growth factor receptor (Rajagopalan et al., 2008), and extracellular signal-regulated kinases (Pantos et al., 2007). THs also influence cardiac mitochondrial function (Marín-García, 2010) and regulate impaired myocardial bioenergetic status and function (Madathil et al., 2015).

In addition, plasma membrane-bound sites include integrin αvβ3, a member of a family of proteins that mediate bidirectional interactions between cells and the extracellular matrix (ECM) and regulate tissue organization and cell migration processes. The integrin αvβ3, which is a member of a family of proteins that interactions between cell and the ECM and regulate migration processes, dependent pathway is primary T4-sensitive and effective some intracellular signals, such as protein kinase B (PKB/AKT) and mitogen-activated protein kinases (MAPKs) that phosphorylate intracellular proteins. In addition, some of them can regulate the nucleus and transcription (Cayrol et al., 2019; Davis et al., 2019; Hercbergs, 2019).

The mitochondrial isoforms of other hormone receptors, including mtRXR (mitochondrial RXR), have also been identified (Casas et al., 2003). In addition of the localization of TR (thyroid receptor) in mitochondria there may be TH-mediated pathway between the nuclear and mitochondrial genome (Wirth and Meyer, 2017).

## Hyperthyroidism and the Cardiovascular System

### Overview of Hyperthyroidism

Hyperthyroidism is commonly affected by stimulation of the TSH receptors by autoantibodies [Graves’ disease (GD)] or as a result of the autonomous production of THs by thyroid nodules (Cooper and Biondi, 2012). In general, the prevalence of hyperthyroidism is approximately 0.5% (Cooper and Biondi, 2012). It predominantly affects women aged 30–50 and is caused by GD in 70% of cases. Graves’ disease with TSH receptor antibodies is characterized by a diffuse goiter, exophthalmos, and pretibial myxedema. Aside from GD, 20% of patients with hyperthyroidism show autonomous production of THs by a nodular goiter (Toft and Boon, 2000).

The cardiac effects in thyroid hormones upregulate resting heart rate, blood volume, and myocardial contractility compared to normal. However, as shown in Table 1, exacerbation of hyperthyroidism can also be detrimental to cardiac function (von Hafe et al., 2019). In patients with hyperthyroidism, exercise intolerance is caused by impaired ability to further increase the heart rate and cardiac contraction and to lower the SVR (Forfar et al., 1982). In a consecutive case series study of 24 patients, Duyff et al. reported that 16 patients showed objective signs or symptoms of neuromuscular dysfunction (Duyff et al., 2000). Cardiac output is markedly elevated in patients with hyperthyroidism. The hyperthyroidism has been implicated in a 16% increase in the risk of major cardiovascular events, as well as involvement in an increase in cardiovascular death (Selmer et al., 2014).

### Hyperthyroidism and Arrhythmias

Cardiac arrhythmias or electrocardiogram (ECG) abnormalities, which include sinus tachycardia, AF, and shortened PR and QT intervals, are sometimes observed in patients with hyperthyroidism (Kahaly and Dillmann, 2005). Although it is rare, atrio-ventricular blockage might be observed in patients with GD (Mohr-Kahaly et al., 1996). In almost all patients with hyperthyroidism, the most common rhythm disturbance is sinus tachycardia (Nordyke et al., 1988; Biondi et al., 2000).

Atrial fibrillation (AF) is recognized as the most common supraventricular arrhythmia in patients with thyrotoxicosis (Nakazawa et al., 2000). In patients with hyperthyroidism, the prevalence of AF ranges between 2 and 20%, and their risk of AF is approximately six-fold higher than that of healthy people (Klein and Danzi, 2007). The primary consideration for the management of AF is to control heart rate. β-blockers are one of the widely used drugs in the treatment of AF in cases of hyperthyroidism (Klein and Danzi, 2007). These drugs can bring down the ventricular rate and stabilize the rapid symptoms, but they have little effect on converting AF to sinus rhythm or on hyperthyroidism. Therefore, treatment of hyperthyroidism is optimal for long-term AF management. This normally employs radioiodine treatment or antithyroid drugs (ATDs), which can restore sinus rhythms within a few months in the majority of hyperthyroidism patients (Nakazawa et al., 2000). One prospective study in middle-aged and elderly people in Rotterdam (Rotterdam Study) reported that the risk of AF, sudden cardiac death, and decreased life expectancy is associated with elevated free T4 levels, even if thyroid function is within normal limits (Bano et al., 2017; Razvi et al., 2018).

While the major arrythmias in patients with hyperthyroidism are atrial, ventricular arrhythmias are rare and occur about as often as in healthy people (Osman et al., 2002). Ventricular tachycardia (VT) is one of the major causes of death in patients with CVD. A cardiac electrical storm (ES) is defined as electrical instability involving hemodynamic disturbances with significant VT, occurring in at least three or more episodes within 24 h, and requiring direct current cardioversion (Dorian and Cass, 1997). This is likely in patients with hyperthyroidism, in whom VT is typically severe (Colzani et al., 2001; Jao et al., 2004). Ventricular arrhythmia is seen in thyrotoxicosis patients undergoing antithyroid therapy, though only rarely (von Olshausen et al., 1989; Osman et al., 2002). Ventricular tachycardia usually occurs in association with underlying structural heart diseases or HF from various etiologies (Polikar et al., 1993; Marrakchi et al., 2015).

### Hyperthyroidism and Heart Failure

Hypothyroidism and hyperthyroidism can both lead to HF (Schmidt-Ott and Ascheim, 2006), and the prognosis for patients with hyperthyroidism even at a mild level is poor, as these patients suffer from arrythmia, as well as HF. When these patients do not receive treatment, hyperthyroidism might lead to HF because of arrhythmias, cardiac hypertrophy, and increased blood volumes (Biondi, 2012). Furthermore, in a case-based study, patients with hyperthyroidism who did not get proper medical treatment had a higher mortality risk with CVD (Franklyn et al., 2005; Vale et al., 2019). Patients with severe hyperthyroidism can suffer from “high-output HF”. Precisely, high-output HF is defined as congestive HF with increasing cardiac output (DeGroot and Leonard, 1970); resulting “tachycardia-induced cardiomyopathy” depends on the duration of high-output HF with hyperthyroidism (Cruz et al., 1990).

In young patients with hyperthyroidism, this thyrotoxicosis is not associated with underlying heart disease, and therefore, the heart is not damaged. However, symptoms of HF occur in cases of enlarged cardiac output and low SVR, and enlarged blood flow volumes caused by chronic stimulation of the renin angiotensin aldosterone system can be present. The symptoms of patients with high-output HF are breathlessness at rest, fatigability, and the accumulation of fluid with peripheral edema, pleural effusion, pulmonary hypertension, and hepatic congestion (Biondi, 2012).

Siu et al. have estimated that the risk of low-output HF is 6–15% in patients with hyperthyroidism (Siu et al., 2007). Elderly patients with hyperthyroidism might suffer from HF with a reduced ejection fraction. These low-output HF patients have low cardiac output, increasing the SVR, a reduction in ventricular contractility, and impaired left ventricular filling, without increased blood volume. The risk of HF with reduced ejection fraction is increased in patients with hyperthyroidism suffering from cardiac disorders such as ischemic heart disease, hypertensive heart, valvular disease and/or AF (Biondi, 2012). Clinically apparent hyperthyroidism with a hyperdynamic state increases the risk of AF (Cooper and Biondi, 2012). The synergistic effects of reducing SVR, increasing contractility, and increasing the heart rate augments cardiac output (Cooper and Biondi, 2012).

It has been reported that cardiovascular diseases related to thyroid function can be further improved by treating the thyroid gland (Barreto-Chaves et al., 2020). Muthukumar et al. reported that patients with cardiovascular dysfunction related to hyperthyroidism could improve their cardiac function by bringing the thyroid to normal levels with thyroid medication (Muthukumar et al., 2016). And they reported that these aforementioned patients could also have their cardiac function completely restored after total thyroidectomy (Muthukumar et al., 2016). In fact, Saad et al. have reported that treatment of the thyroid gland significantly prevented cardiac dysfunction in a mouse model of T4-induced cardiac dysfunction (Saad et al., 2017). Furthermore, reversibility of heart disease was observed after 2 weeks of T4 treatment, including cardiac hypertrophy (Saad et al., 2017).

In SardiNIA study, Delitala et al. demonstrated that serum FT4 levels are associated with carotid-femoral artery PWV (pulse wave velocity), and high levels of free T4 was is one aggravating factor of aortic stiffness. And they considered that T4 may contribute to the atherosclerosis and the aging process in the vascular system (Delitala et al., 2015; Vale et al., 2019).

Importantly, death from heart failure is the major cause of cardiovascular death in both hyperthyroidism and subclinical hyperthyroidism (Selmer et al., 2014).

### Subclinical Hyperthyroidism

Subclinical hyperthyroidism is defined as a condition in which serum TSH levels are below the lower limit of normal, but the width of serum T3 and T4 concentrations is within the normal range (Surks et al., 2004; Biondi and Cooper, 2008; Bahn et al., 2011). Subclinical hyperthyroidism has two major causes. The first encompasses exogenous factors such as an excessive dosage of thyroid hormone replacement drugs, high-dose glucocorticoids, and others. The second is an endogenous factor, namely underlying thyroid disease that causes the overactivity of THs. A considerable proportion (15–20%) of patients who take levothyroxine have a low TSH serum level (Canaris et al., 2000; Vadiveloo et al., 2011; Taylor et al., 2014). In the USA, the prevalence of endogenous subclinical hyperthyroidism varies and depends on age, sex, and iodine intake. Cappola et al. reported that the prevalence of subclinical hyperthyroidism in iodine-sufficient cases is almost 2% (Cappola et al., 2006).

Several observational clinical studies have reported a relationship between subclinical hyperthyroidism and incident CVD (Parle et al., 2001; Iervasi et al., 2007), AF (Cappola et al., 2006; Collet et al., 2012), HF (Rodondi et al., 2008; Biondi et al., 2015), and cardiovascular mortality (Collet et al., 2012). It is not clear whether subclinical hyperthyroidism is associated with high-risk cardiovascular morbidity and mortality. However, the European Thyroid Association guidelines recommend that older patients with hyperthyroidism should have a serum TSH < 0.1 mU/L (Biondi et al., 2015).

### Treatment of Hyperthyroidism

In this section, we review the medical history of GD, which is a major cause of hyperthyroidism. Graves’ disease is treated by ATDs, which decrease TH synthesis, by radioactive iodine (RAI) therapy, or total thyroidectomy (Smith and Hegedüs, 2016; Kahaly et al., 2018). Antithyroid drugs is the major treatment in Europe, United States, and Asia (Brito et al., 2016). The main ATDs are thionamides, for example propylthiouracil (PTU), carbimazole (CBZ), and methimazole (MMI). Propylthiouracil controls the conversion from T4 to T3 by inhibiting enzymatic activity in the peripheral organs such as the liver or kidney. As a result, PTU further reduces the density of blood T3. Carbimazole acts by obstructing hormone generation in the thyroid gland, and more significantly, by affecting the hyperstimulation of the thyroid gland at a level prior to biosynthesis. Carbimazole must be decarboxylated to produce MMI in the liver. Methimazole, as well as PTU, is absorbed immediately and accumulates at a high density in the thyroid gland, restraining the synthesis process of TH. All thionamides inhibit the coupling of iodothyronines and reduce the biosynthesis of THs (Cappola and Ladenson, 2003). As a result, these drugs inhibit the production of TH by iodide peroxidase. Specifically, iodide peroxidase oxidizes an iodide ion to iodine and iodinates a tyrosine residue of the thyroglobulin, and this process is indispensable in the production of T4. ATD has been recommended as a first choice drug for the treatment of GD, particularly for short-period GD treatment prior to thyroidectomy or RAI therapy (Bartalena, 2013; Smith and Hegedüs, 2016). Higher doses of PTU inhibit the deiodination of T4 to T3 (Cooper, 2005) and have severe side effects, such as severe hepatic disorder and anti-neutrophil cytoplasmic antibodies (ANCA)-associated vasculitis. However, the half-life of PTU (75 min–150 min) is much shorter than that of MMI (6 h) (Kahaly et al., 2018). Hyperthyroidism is linked to risk of enlargement in CVD and mortality in the first year following radioiodine treatment, but early diagnosis and proper treatment of hyperthyroidism along with cardiac treatment might reduce mortality (Toft and Boon, 2000).

## Hypothyroidism and the Cardiovascular System

### Overview of Hypothyroidism

In general, hypothyroidism is diagnosed when serum TSH levels are high (usually > 10 mU/L) and serum-free T4 levels are low (< 9–10 pmol/L). Clinically apparent hypothyroidism can be seen in 0.2–2.0% of non-pregnant adults (Canaris et al., 2000). Symptomatic thyroid dysfunction is found in 1–2% of the population and is more frequent in women. Except for previous radioiodine treatment or thyroidectomy for GD, the most common cause is autoimmunity of the thyroid gland or Hashimoto’s thyroiditis (Hashimoto’s disease). Hashimoto’s thyroiditis can often be accompanied by hard goiter, in which the thyroid gland becomes atrophied causing progressive fibrosis during the course of the disease, and resulting in diminished function of the thyroid gland. Distinct from hyperthyroidism, there is a link between low serum concentrations of HTs (T3 and T4) and a reduction in cardiac output, heart rate, stroke volume, and myocardial contractility (Toft and Boon, 2000).

One of the most important cardiac dysfunctions, especially among patients with hypothyroidism, is diastolic dysfunction. This diastolic dysfunction is observed not only at rest, but also during exercise. This exacerbates the symptoms and worsens the prognosis of potential heart failure patients in hypothyroid patients (Selmer et al., 2014; von Hafe et al., 2019). It is well known that hypothyroidism is associated with chronic heart failure. However, in rare cases, hypothyroidism may also be associated with pericardial effusion and cardiac tamponade (Grais, 2010; Patil et al., 2011).

In addition to the abovementioned clinical symptoms, remarkable changes in modifiable atherosclerotic risk factors are also observed in clinically apparent hypothyroidism, including hypercholesterolemia, diastolic hypertension, carotid intima-media thickening, and reduced production of endothelial-derived relaxation factor (nitric oxide) (Cappola and Ladenson, 2003). All of these clinical manifestations are improved by TH replacement therapy (Cappola and Ladenson, 2003).

### Hypothyroidism and Arrhythmias

It is generally accepted that typical hypothyroid ECG changes include bradycardia, long-PQ segment, low voltage of the QRS complex, and flattening or T-wave inversion. However, it is important, though not well known, that hypothyroidism induces atrioventricular blockage and acquired long QT syndrome (Marrakchi et al., 2015). In the case of supraventricular arrhythmias, hypothyroidism or subclinical hypothyroidism has a certain clinical impact. For example, patients with thyroid dysfunction have a higher probability of having AF than healthy people (Sawin, 1995; Baumgartner et al., 2017). Klemperer et al. reported that perioperative T3 treatment reduces the incidence or necessity of postoperative AF in patients with normal thyroid function during cardiopulmonary bypass surgery. However, experiments on the mechanism underlying this discovery have not yet been reproduced (Klemperer et al., 1996). Kim et al. confirmed that hypothyroidism has no relationship with 10-year risk of incident AF from the famous cardiovascular cohort of the Framingham Heart Study (Kim et al., 2014).

Regarding ventricular arrhythmias, some groups have reported that patients with hypothyroidism might experience life-threatening arrhythmia, for example a torsade de pointes type ventricular tachycardia (TdP type ventricular tachycardia) and VT due to prolonged QT syndrome (Chojnowski et al., 2007). That study also reported that hypothyroidism decreases the expression of protein T3 in cardiomyocytes and that it can cause reduced cardiac contractility and heart rate, as well as delayed conduction of electrical stimulation in the heart. This might be the reason for bradycardia and elongation of the QT interval and consequent fatal arrhythmia, such as TdP-type ventricular tachycardia. In this case, the causes of long QT syndrome and shock include the decreased T3 expression and disorders of the electrolyte balance, for example hypokalemia and hypocalcemia (Chojnowski et al., 2007). For patients with hypothyroidism, it is necessary to monitor the effectiveness of amiodarone for the prevention of ventricular arrhythmic recurrence. It has been reported that lidocaine (class IB antiarrhythmic drug) or bretylium tosylate (class III antiarrhythmic drug) might be useful to prevent these paroxysmal ventricular tachycardias and endocavitary electrode stimulation, in place of amiodarone (Chess-Williams and Coker, 1989).

### Hypothyroidism and Heart Failure

It is thought that TH deficiency raises the risk of developing and exacerbates HF (Rodondi et al., 2008). Basic experiments have reported that hypothyroidism suppresses myosin heavy chain 6 protein expression and enhances myosin heavy chain 7 protein expression and that hypothyroidism induces cardiac atrophy as a result. In addition, hypothyroidism is associated with the increased dilation of ventricular chambers and reduced myocardial perfusion (Liu et al., 2008; Biondi, 2012). Congestive HF and myxedema have been recognized in patients with hypothyroidism (Schwimmer et al., 1947), and their HF and myxedema symptoms improved with treatment for hypothyroidism. More recently, it was reported that patients with hypothyroidism were among patients with reversible dilated cardiomyopathy (Khochtali et al., 2011). Recent clinical studies have reported that patients with cardiovascular disease who have reduced T3 levels have a higher risk of death from heart failure (Wang et al., 2017; Neves et al., 2019). Rezvi et al. reviewed thyroid hormone supplementation in HF (Neves et al., 2020). Studies in patients without HF as well as HFrEF suggest an effect of thyroid hormone supplementation in improving diastolic function (Pingitore et al., 2008). In the HFrEF animal model, it has been suggested that thyroid hormone supplementation in HF improves cardiac function (Vale et al., 2019).

It is interesting that the metabolism and serum levels of THs are changed by conditions of HF, myocardial infarction, and cardiac surgery. In these situations, the conversion of T4 to T3 decreases. Diseases with normal serum levels of TSH and no symptoms suggestive of hypothyroidism despite a decrease in blood thyroid hormone are called euthyroid sick syndrome or non-thyroidal illness. Among them, those with only low T3 levels are called low T3 syndrome (LT3S) (Nagayo, 2018).

Amin et al. reported that the Patients with heart failure with HFrEF (left ventricular ejection fraction < 40%) and with LT3S take the oral T3 supplements (Liothyronine) for 1.5 months, and that the patients can be found to improve cardiac function considering the blood laboratory data and echocardiographic data (Liothyronine group *N* = 25, Placebo grouper *N* = 25) (Amin et al., 2015). Further, LT3S impairs cardiac dysfunction and as a result induces heart disease. Patients with advanced heart disease and LT3S have increased mortality (Pingitore et al., 2005; Gerdes and Iervasi, 2010; Mourouzis et al., 2011).

Pingitore et al. conducted a clinical study to determine whether T3 administration improves cardiac function in patients with low T3 syndrome (LT3S) who have suffered an acute myocardial infarction (AMI) (LT3S/AMI) (Pingitore et al., 2019). Pingitore et al. concluded that the patients with LTS3S/AMI had improved cardiac functions, which were assessed using cardiac MRI to evaluate various parameters (for example infarct sizes, and cardiac function), after 6 months of treatment with liothyronine (T3) therapy [The THIRST Study (Thyroid Hormone Replacement Therapy in ST elevation myocardial infarction); Phase II study] (T3 supplement group *N* = 19, Placebo grouper *N* = 18) (Pingitore et al., 2019; Lisco et al., 2020). Some researchers reported that the changes in gene expression associated with cardiac dysfunction are similar to those induced in hypothyroidism, suggesting that TH dysfunction might be one aggravating factor for HF (Kinugawa et al., 2001; Biondi, 2012).

### Hyperlipidemia and Coronary Artery Disease in Hypothyroidism

THs are involved in lipid metabolism (Cappola and Ladenson, 2003). Hyperthyroidism is not an exacerbating factor for the lipid profile. For several years, hypothyroidism was thought to be linked to hyperlipidemia. In fact, many patients with clinical hypothyroidism show obvious clinical symptoms (Klein and Danzi, 2007; Jabbar et al., 2017).

There are two aspects of the association between hypothyroidism and coronary disease. First, hypothyroidism has an influence on hypertension and hypercholesterolemia in these patients. In particular, this hypertension and hypercholesterolemia due to hypothyroidism accelerate atherosclerosis. Second, it is thought that hypothyroidism reduces cardiac oxygen requirements and decreases their effective use and that this process induces CVD (Kahaly and Dillmann, 2005). It is notable that low serum T4 levels are associated with increased low-density lipoprotein-cholesterol and that hypothyroidism has also been implicated in hyper-triglyceridemia and low free fatty acid levels.

Thyroid hormone replacement is desirable in patients with hypothyroidism, even in patients with myocardial infarction. In fact, this is because thyroid hormone is said to be an important factor in regulating the structure and function of the left ventricle in the late post-myocardial infarction period (Jankauskienė et al., 2016; von Hafe et al., 2019). In molecular biology, low T3 levels can induce oxidative stress and apoptosis, which may exacerbate ventricular dysfunction (Jankauskienė et al., 2016).

Zhang et al. reported that in a rat model of cardiac reperfusion injury, the addition of T3 enhances the gene expression of transcription factor Hypoxia-Inducible Factor alpha (HIF-1α). It thereby regulates mitochondrial opening and protects cardiomyocytes (Zhang et al., 2018).

### Subclinical Hypothyroidism

Subclinical hypothyroidism is defined as being associated with a serum TSH level above the normal range, but with normal TH levels. Subclinical hypothyroidism is classified into two types according to the level of TSH. Mild subclinical hypothyroidism is diagnosed by a mildly high TSH level (between 4.0–4.5 and 10.0 mU/L). Severe subclinical hypothyroidism is diagnosed by a TSH level > 10.0 mU/L. However, the upper limit of normal TSH has not been clearly defined. For the diagnosis of subclinical hypothyroidism, it is necessary to consider both clinical symptoms and the level of TSH (Hamilton et al., 2008; Razvi et al., 2018). In general, the prevalence of subclinical hypothyroidism is 4–20% in adults (Biondi and Cooper, 2008; Abreu et al., 2017). There are many causes for this wide range of reported prevalence. Specifically, differences in age, sex, race, body mass index, dietary iodine intake, and serum TSH measurements at different diagnostic institutions might contribute to this. The prevalence of a high serum level of TSH is higher in Caucasians than in African-American people (Hollowell et al., 2002). It is also thought that at least 10% of older women (aged 60 and older) are diagnosed with subclinical hypothyroidism (Parle et al., 1991). This wide prevalence suggests that patients with more cardiovascular risk factors than expected are present. The cardiac function of patients with subclinical hypothyroidism shows abnormalities including extended isovolumic relaxation time and impaired ventricular filling (Monzani et al., 2001). To date, several studies of systolic dysfunction in patients with subclinical hypothyroidism have been conducted. Ripoli et al. reported that there is an association between subclinical hypothyroidism and systolic dysfunction and that thyroxine replacement therapy improves cardiac contractility in patients with hypothyroidism (Ripoli et al., 2005). In patients with subclinical hypothyroidism during exercise, both diastolic and systolic function are impaired and as a result, exercise tolerance is reduced in these patients (Brenta et al., 2003).

It is not currently known whether there is an association between the severity of subclinical hypothyroidism and increased risk of CVD. According to meta-analyses of observational studies, patients aged < 65 years (Razvi et al., 2008) with severe hypothyroidism, defined as a TSH level > 10 mU/L, do have a high risk of CVD (Rodondi et al., 2010). According to the guidelines of the European Thyroid Association, treatment for hypothyroidism is recommended for patients with severe hypothyroid disease (serum THS > 10 mU/L), symptoms of hypothyroidism, an age < 70 years, and elevated risk of CVD (Pearce et al., 2013).

### Treatment of Hypothyroidism

One favored therapy involves taking levothyroxine (L-thyroxine) with solid preparation on an empty stomach. Patients who not only have clinical manifestations of hypothyroidism, but also a diagnosis based on biochemical examinations, are recommended treatment. Levothyroxine is marketed by several pharmaceutical companies, but switching to generic levothyroxines is not recommended for patients who are stable with one regimen (Jonklaas et al., 2014; Chaker et al., 2017). In patients with clinically manifesting hypothyroidism, 1.5–1.8 mg/kg/day of levothyroxine is desirable as a daily optimal dose (Pearce et al., 2013; Jonklaas et al., 2014). Generally, in patients with CVD, the initial dose is 12.5–25.0 mg/day. Based on symptoms and the serum TSH level, it is desirable to gradually increase the dose afterward (Jonklaas et al., 2014).

Thyroid hormone replacement therapy improves diastolic function in patients with hypothyroidism and also in patients with heart failure with preserved ejection fraction (HFpEF). Thyroid hormone is said to improve cardiac diastolic function, both pathophysiologically and through gene expression. However, further preclinical and clinical studies are needed to clarify the role of thyroid hormones in the treatment of HFpEF (Neves et al., 2020). In patients with HFpEF, it is advisable to use beta-blockers in combination with thyroid hormones when they are used. The risk of cardiovascular events due to sympathetic hyperactivity in thyroid hormones can be inhibited by the combined use of beta-blockers. As a result, the combination of the two drugs can improve cardiac function while foreshadowing arrhythmias, cardiac hypertrophy, and cardiac dysfunction (Ortiz et al., 2019).

For some CVD patients with hypothyroidism, cardiac function is improved by hypothyroidism treatment. Furthermore, treatment can improve prognostic factors of thyroid function and the cardiovascular system (Crowley et al., 1977). However, it has not been clarified whether patients treated for hypothyroidism have exacerbated heart disease when the treatment is discontinued (Klein and Danzi, 2007). The clinical implications of low T3 levels in patients with normal TSH levels have also not yet been determined. Measurement of T3 does not correlate with therapeutic efficacy (Abdalla and Bianco, 2014).

## Drug-Induced Thyroid Dysfunction

### Effects of Cardiovascular Drugs on Thyroid Function

The interrelationship between thyroid hormones and the heart is a very important focus area. Thus far, we have described how changes in THs cause cardiovascular dysfunction. In this section, we summarize the ways in which cardiac medications routinely used by cardiologists affect thyroid hormones.

As mentioned above, among drugs used commonly by many cardiologists, there are some that alter TH levels in patients with normal thyroid function. Several of these can alter the levels of THs in euthyroid patients. Fadel et al. summarized the effects of cardiovascular drugs on thyroid function [Table 3, (Fadel et al., 2000)]. When cardiovascular drugs are used, TH levels must be checked every 3–4 months. The most widely used drug that affects thyroid function is amiodarone. Approximately 50% of patients taking amiodarone for a long period have elevated serum T4 levels. However, the serum levels of T3 and TSH are within a normal range (Harjai and Licata, 1997). One of the main effects of amiodarone on thyroid function is the inhibition of the conversion of T4 to T3 by amiodarone and its major active metabolite, desethylamiodarone (Burch, 2019). Ruzieh et al. reported that the likelihood of experiencing an amiodarone-related adverse event was greater than with placebo. (relative risk about 4.44 versus placebo) (Ruzieh et al., 2019). In the following sections (6.2 to 6.4), we summarize the effect of amiodarone on thyroid function in detail.

**TABLE 3:** Effect of cardiovascular drugs on thyroid hormone function [modified from reference (Fadel et al., 2000; Burch, 2019)].

Drugs	Medical effect	Thyroid hormone serum concentrations			Mechanism of action in the thyroid	References
		TSH	T3	T4		
Amiodarone (∼3months)	Class III antiarrhythmic drug	↑	↓	↑	Inhibition of conversion of T4 toT3 inhibition the biosynthesis and release of TH	Harjai and Licata, 1997; Martino et al., 2001; Burch, 2019
Amiodarone chronic therapy (chronic phase: 3 months ∼)	Class III antiarrhythmic drug	→	→ or ↓	↑	Inhibition of T4 to T3 conversion Inhibition the biosynthesis and release of TH	Harjai and Licata, 1997; Martino et al., 2001; Burch, 2019
Dopamine	Adrenergic drug	↓	→	→	Suppression of TSH production	Cooper et al., 1983; Fadel et al., 2000
Furosemide (high dose)	Loop diuretic drug		↓	↓, slight ↑free T4 ?	Inhibition of T3/T4 binding to TBG	Fadel et al., 2000; Burch, 2019
Heparin (IV)	Anticoagulant drug			slight ↑, slight ↑ free T4	Inhibition of T4distribution in targeted tissue	Mendel et al., 1987; Burch, 2019
Propranolol (high dose)	β1 Non-selective blocker			↑, ↑free T4	Inhibition of T4 uptake in targeted tissue	Shenfield, 1981; Burch and Wartofsky, 2018; Burch, 2019

*IV; intravenous administration, TGB; thyroxine binding globulin, ↑; increase, ↓; decrease, →; unchange, ?; unknown.*

This section summarizes the relation between some drugs in cardiology and thyroid function.

Dopamine suppressed TSH secretion in about 50% of humans. Administration of the dopamine receptor antagonists metoclopramide or domperidone increases TSH in primary hypothyroidism. Normal level of THs suppresses TSH elevation, but hypothyroidism does not respond to this suppression, resulting in an elevation response (Cooper et al., 1983). High dose fulosemide is said to attenuate the effects of thyroid hormones by binding to TGB (thyroxine binding globulin), which is one of transporter portein of THs (Fadel et al., 2000; Burch, 2019). However, there are few documents that explain the above in pharmacokinetics, and it is necessary to examine it in detail in the future. Heparin increases T3 and T4 from binding proteins indirectly by increasing free fatty acids (Mendel et al., 1987; Burch, 2019). Propranolol in high volume inhibits the conversion of T4 to T3. As a result, it increases T4 and free T4 (Burch and Wartofsky, 2018; Burch, 2019). In hyperthyroidism, the effect of propranolol is enhanced (Shenfield, 1981), and vice versa in hypothyroidism (Burch, 2019).

### Overview of Amiodarone and Thyroid Function

Amiodarone is regarded as the most effective antiarrhythmic drug but has various toxicities, and cardiologists need to exercise caution (Trohman et al., 2019). The combination of amiodarone and a beta-blocker is the preferred treatment for ES (Nademanee et al., 2000). According to the 2017 AHA/ACC/HRS Guidelines for Management of Patients with Ventricular Arrhythmias and the Prevention of Sudden Cardiac Death, amiodarone is recommended as a Class IIb indication for the acute treatment of hemodynamically stable VT (Al-Khatib et al., 2018). Amiodarone is effective at suppressing AF, as well as ventricular arrhythmia. In particular, it is thought to be the most effective drug for maintaining sinus rhythms against paroxysmal and persistent AF for a long duration. It is also recognized as the first-choice agent for patients with AF due to HF or symptomatic AF (Singh, 2008; Al-Khatib et al., 2018).

The numerous side effects of amiodarone might involve the skin, eyes, brain, lungs, liver, and peripheral nervous system (Trohman et al., 2019). The major side effect is the production of corneal microdeposits (> 90% of treated patients). These are very similar in appearance to vortex keratopathy, which is associated with Fabry disease (D’Amico et al., 1981; Wasielica-Poslednik et al., 2011).

It is important for clinical physicians, as well as cardiologists, to keep in mind that the use of amiodarone affects thyroid function. That is, some patients using amiodarone suffer from hypothyroidism (5% to 10% of treated patients) and some suffer from hyperthyroidism (0.9–10% of treated patients) (Harjai and Licata, 1997; Trohman et al., 2019). The iodine richness of amiodarone is the recognized cause of its thyrotoxic side effects.

Changes in serum TSH, T4, and T3 levels are seen in patients receiving amiodarone treatment. The pattern of TH levels differs between the acute phase (∼ 3 months) and the chronic phase (after 3 months) after the start of amiodarone use. In both the acute and chronic phases, the serum T4 level rises and the serum T3 level decreases, though the serum reverse T3 (rT3) level rises. Interestingly, the serum TSH levels are elevated in the acute phase, but return to a normal range during the chronic phase (Martino et al., 2001). There are two reasons why serum TH levels change during these two phases, specifically (1) the unique effects of amiodarone and (2) the effects of its constituent iodine (Basaria and Cooper, 2005).

### Amiodarone-Induced Hyperthyroidism

Amiodarone can cause hyperthyroidism (amiodarone-induced thyrotoxicosis: AIT), which is subdivided into two main forms. Type I AIT is usually a predisposing factor for goiter abnormalities (for example asymptomatic GD) and results from the over-synthesis and over-release of THs. The self-regulatory mechanism of THs regulates iodine metabolism in the thyroid gland according to iodine content (Harjai and Licata, 1997). Typical cases of type I AIT occur in patients with a background of non-toxic goiter or asymptomatic GD with an overdose of iodine (Trohman et al., 2019). The prevalence of type I AIT is higher in areas with low iodine intake. Treatment for AIT type I is similar to that for normal hyperthyroidism (Hamilton et al., 2020).

Type II AIT is a thyrotoxicosis cause by destructive thyroiditis and is characterized by acute or subacute destruction of the thyroid gland and massive leakage of THs in patients with no underlying thyroid disease while taking amiodarone (Tsang and Houlden, 2009). The prevalence of type II AIT is higher in areas with high iodine intake. Mild thyroiditis of type II AIT (with mild elevation of free T4 and free T3) is likely to improve spontaneously with follow-up alone. In severe type II AIT, the use of glucocorticoid is beneficial even under amiodarone administration (Daniels, 2001; Hamilton et al., 2020).

Sometimes we find the patients who have a combination of the two types of AIT. In such cases, it is advisable to treat them in both types of AIT. Specifically, type 1 should be treated with antithyroid therapy and type 2 with steroids (Osuna et al., 2017; Omidi et al., 2020).

### Amiodarone-Induced Hypothyroidism

Amiodarone can also cause hypothyroidism, which inhibits the biosynthesis and release of THs. This is called amiodarone-induced hypothyroidism. This is because large amounts of iodine are released during amiodarone metabolism (Harjai and Licata, 1997). Intrathyroid iodine organization resumes with the normal synthesis of T4 and T3 (Martino et al., 1984). The development of hypothyroidism with amiodarone has been associated with previous Hashimoto’s thyroiditis. However, the use of amiodarone is not contraindicated even if serum TSH levels are elevated before or during treatment, because thyroid dysfunction can be easily treated with T4 (Toft and Boon, 2000). After discontinuing amiodarone treatment, thyroid function might recover, but persistent reduced function has been observed. In particular, many patients for whom thyroid function does not improve even after the discontinuation of oral amiodarone administration are considered to have underlying autoimmune thyroid (Martino et al., 2001).

## Conclusion

In this manuscript, we discussed how TH plays an important role in cardiovascular disease at the molecular level via genomic and non-genomic pathways. Serious cardiac complications such as arrythmia, congestive HF, and angina pectoris might arise in patients with hyperthyroidism or hypothyroidism, and their treatment requires control of the underlying TH levels. Judging from previous clinical observational studies and small-scale intervention studies, which have evaluated the association between THs and risk factors for CVD, it can be concluded that among patients who should be treated for thyroid function, those with severe CVD should be given priority thyroid treatment (Biondi and Cooper, 2008). Abnormal levels of THs might also serve as a prognostic marker (Jabbar et al., 2017).

Since the discovery of iPS cells by Takahashi and Yamanaka (2006), the study of myocardial regeneration has attracted the attention of scientists worldwide (Takahashi and Yamanaka, 2006). Several hormones have been suggested to be involved in myocardial regeneration. One of these is TH, which was recently reported to affect both the metabolic profile and the mitotic cycle in cardiomyocytes (Nakada et al., 2017; Hirose et al., 2019; Tan et al., 2019; Bogush et al., 2020).

In mouse cardiomyocytes, prolonged inhibition of TH with propylthiouracil prolongs the proliferative period of cardiomyocytes during cardiac development (Hirose et al., 2019). Transgenic mice with cardiac-specific suppression of TRα exhibited a higher number of cardiomyocytes and higher expression of cell cycle markers compared to normal mice during cardiac development. In addition, adult TRα transgenic mice demonstrated a 10-fold increase in the number of proliferating diploid cardiomyocytes, as well as improved recovery of cardiac function after ischemia-reperfusion injury (Hirose et al., 2019).

Two recent reports have shown that exogenous administration of thyroid hormone (T3) increases the number of cardiomyocytes in neonatal mouse hearts (Tan et al., 2019; Bogush et al., 2020). Tan et al. determined that T3 activates mitochondria-generated H_2_O_2_ (mH_2_O_2_), which in turn activates c-Jun N-terminal kinase-2α2 (JNK2α2) via peroxiredoxin-1 (Tan et al., 2019) to promote cardiomyocyte proliferation. Exogenous T3 stimulates proliferative ERK1/2 signaling and affects cardiomyocytes in the cardiac apex, but is suppressed in P8 by the expression of DUSP5, a nuclear phosphho-ERK1/2-specific dual-specificity phosphatase (Bogush et al., 2020).

It is important to maintain optimal levels of THs in the cardiac tissue, as well as suitable serum TH levels, to stabilize homeostasis. Further, basic studies (such as molecular biology and omics-based analysis), and clinical studies (such as cohort studies and randomized clinical trials) of THs will help us understand their effects on CVD. Moreover, we can elucidate the mechanisms of the demonstrated effects via induced arrhythmia or myocyte remodeling and dysfunction.

## Author Contributions

HY and YA: conceptualization and funding acquisition. HY: writing – original draft preparation. TK and YA: writing – review and editing. HY, SY, and KF: supervision. All authors contributed to the article and approved the submitted version.

## Conflict of Interest

The authors declare that the research was conducted in the absence of any commercial or financial relationships that could be construed as a potential conflict of interest.

## Publisher’s Note

All claims expressed in this article are solely those of the authors and do not necessarily represent those of their affiliated organizations, or those of the publisher, the editors and the reviewers. Any product that may be evaluated in this article, or claim that may be made by its manufacturer, is not guaranteed or endorsed by the publisher.

## Abbreviations

AF: atrial fibrillation
AIT: amiodarone-induced thyrotoxicosis
AKT: serine/threonine-protein kinase
AMI: acute myocardial infarction
ANCA: anti-neutrophil cytoplasmic antibodies
ATD: antithyroid drugs
CBZ: carbimazole
CVD: cardiovascular disease
DUSP5: specific dual-specificity phosphatase 5
EC coupling: excitation-contraction coupling
ECG: electrocardiogram
ECM: extracellular matrix
ERK: extracellular signal-regulated kinase
ES: electrical storm
GD: Graves’ disease
HF: heart failure
HFpEF: heart failure with preserved ejection fraction
HFrEF: heart failure with reduced ejection fraction
HIF-1α: Hypoxia-Inducible Factor alpha
IGF-1: insulin-like growth factor-1
IV: intravenous administration
JNK2α2: c-JunN-terminal kinase-2 α 2
LT3S: low T3 syndrome
MAPK: mitogen-activated protein kinase
MMI: methimazole
MMP: matrix metalloproteinase
mH_2_O_2_: mitochondria-generated H_2_O_2_
mtRXR: mitochondriala RXR
MYH: myosin heavy chain
NCX: Na + /Ca2 + exchanger
PI3K: phosphatidylinositol 3-kinase
PKB: protein kinase B
PLN: phospholamban
PTU: propylthiouracil
PWV: pluse wave velocity
RAI: radioactive iodine
RXR: retinoid X receptor
SCD: sudden cardiac death
SERCA2: sarcoplasmic/endoplasmic reticulum calcium ATPase 2
SVR: systemic vascular resistance
SVR: synergistic effects of reducing
TdP: torsade de pointes
TGB: thyroxine binding globulin
TH: thyroid hormone
TR: thyroid hormone receptor
TRE: thyroid hormone response element
TRH: thyrotropin-releasing hormone
VT: Ventricular tachycardia.
